# A hands‐on introduction to medical physics and radiation therapy for middle school students

**DOI:** 10.1002/acm2.12569

**Published:** 2019-03-18

**Authors:** Jessica M. Fagerstrom, Wendy Gao, Gene E. Robertson

**Affiliations:** ^1^ Northwest Medial Physics Center Lynnwood WA USA; ^2^ Tacoma Valley Radiation Oncology Centers Puyallup WA USA

**Keywords:** community outreach, education, outreach, public education, STEM education, teaching

## Abstract

Lesson plans were developed to present concepts of medical physics and radiation therapy to a middle school audience. These workshop learning units relied on hands‐on participation and collaboration within student groups to acquaint students with computed tomography simulation and treatment planning processes. These lesson plans were delivered at two different educational outreach programs targeted at student groups that have traditionally been underrepresented in science, technology, engineering, and mathematics (STEM) fields. The lesson plans are scheduled to be delivered at a third program in the future. The activities were used to introduce occupations in medical physics and radiation therapy as possible career opportunities for students, and to generate enthusiasm for continuing STEM education. Lesson plans are available upon request for educators interested in exploring medical physics educational outreach activities in their communities.

## 1 INTRODUCTION

A hands‐on introduction for middle school students to careers in therapeutic medical physics and radiation therapy was designed as a way to build interest in careers in science, technology, engineering, and mathematics (STEM). In particular, it was hoped to reach members of student groups that have traditionally been underrepresented in STEM fields. The National Science Foundation^1^ and a joint report from the U.S. Office of Science and Technology and U.S. Office of Personnel Management^2^ define these groups as including women, African Americans, Hispanic Americans, Pacific Islanders, American Indians or Alaska Natives, and people with disabilities.

The American Association of Physicists in Medicine (AAPM) reports a membership of approximately 25% women and 75% men^3^ with 6% of currently employed medical physicists identifying as members of underrepresented minority groups.^4^, ^5^ The AAPM has prioritized efforts to increase equity, diversity, and inclusion, with an emphasis on outreach. The organization maintains an official diversity statement that recognizes that a diverse community of medical physicists allows the AAPM to excel in the clinic, in education, and in research.^6^ The statement asserts that the AAPM, “strive[s] to increase the diversity of the AAPM through outreach and mentoring.” Furthermore, the AAPM recently updated its Focus Areas and Strategic Goals to include an education strategic goal (to “cultivate excellence in medical physics education”) as well as a diversity and inclusion strategic goal [to “champion equity, diversity, and inclusion (EDI) in the field of medical physics”]. With these goals in mind, it was desired to create and deliver a hands‐on learning experience to introduce members of student groups underrepresented in STEM fields to clinical therapeutic medical physics and radiation therapy. Because middle school is a critical time period for student engagement in STEM,^7^, ^8^, ^9^ these lesson plans were developed for a middle school learning audience.

## 2 MATERIALS AND METHODS

Lesson plans were created for two programs in the Seattle metropolitan area: the Burke Museum of Natural History and Culture's “Girls in Science” Program, and the University of Washington‐Bothell's “Inspire STEM” Festival. The Inspire STEM Festival is supported by locally based organizations including the Boeing Company, and the Girls in Science Program is supported in part by a grant from the National Science Foundation. Both of these programs are designed for students in grades 6–8, with attendance especially encouraged for members of student groups traditionally underrepresented in STEM, including girls, minorities, students from low‐income families, and possible future first‐generation college students.^10^ The workshops used overlapping content and learning materials. These lesson plans were prepared as an introduction to medical physics and radiation therapy using activities that are straightforward to implement with inexpensive classroom materials (all required equipment was donated or readily available at local hardware and art supply stores). Learner‐centered activities^11^, ^12^, ^13^ were developed to promote student engagement in solving problems, to give students control over the learning process, and to avoid the traditional active teacher/passive student roles. These lesson plans were designed to foster active learning environments,^14^ requiring students to engage in hands‐on participation, collaboration, and high levels of Bloom's taxonomy of learning.

For both the Inspire STEM Festival and the Girls in Science workshops, students were divided into small groups to complete tasks as teams. Two separate activities were developed: a treatment planning workshop and an immobilization and localization workshop. For the Girls in Science Program (a 3‐h workshop format), both activities were completed. For the Inspire STEM Festival (a 45‐min workshop format), only the treatment planning activity was completed. For both programs, a short PowerPoint presentation (Microsoft Corporation, Redmond, WA) was delivered prior to beginning the hands‐on activities. This presentation included a brief introduction to important concepts for the workshops, including the ionizing energy range of the electromagnetic spectrum, linear accelerators, modern radiation therapy department workflow, and the career path of a clinical medical physicist. During the presentation, examples of decommissioned physics dosimetry equipment were demonstrated and circulated around the classrooms for student inspection. These props included TLDs, survey meters, radiochromic film segments, and personnel dosimeters.

### 2.A Immobilization and localization activity

**Figure 1:**
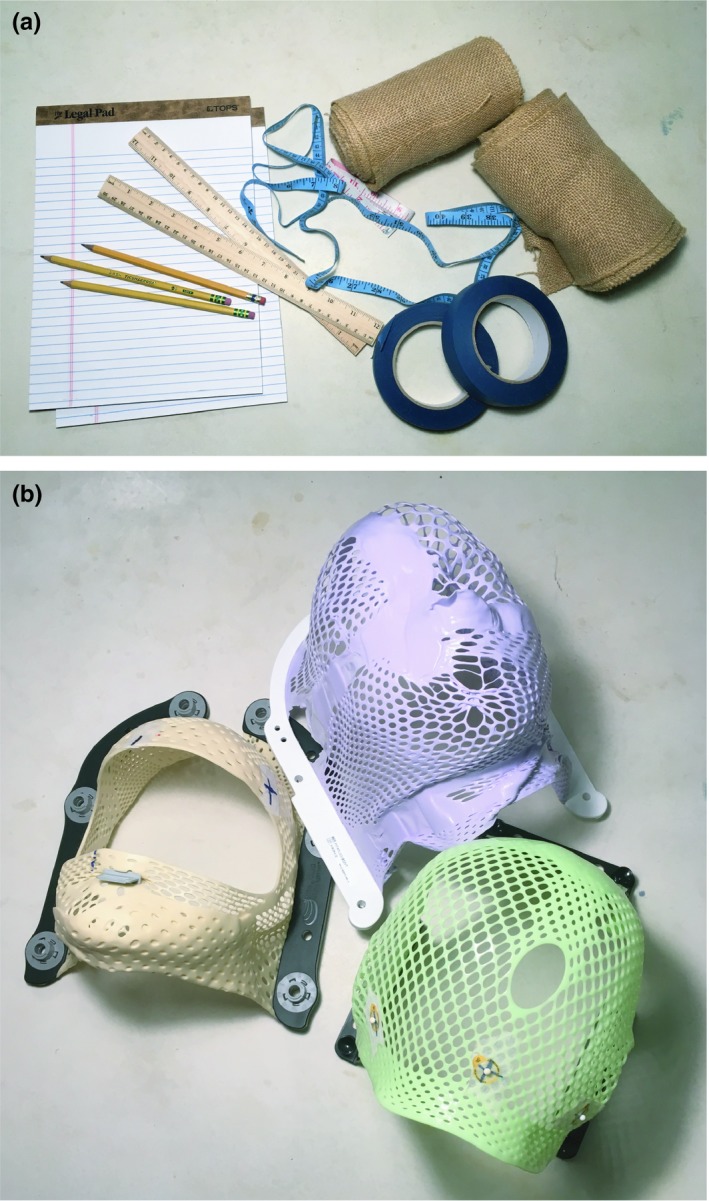
Materials and props used for the immobilization and localization activity, including (a) rulers, fabric tape measures, rolls of painter's tape, fabric cut at four‐meter intervals, pencils, and paper, and (b) a variety of thermoplastic masks used in radiation therapy for positioning patients being treated in the head or neck region.

The first activity was designed to introduce students to practical considerations of computed tomography (CT) simulation. For this activity, each student group was provided with simple materials including a ruler, fabric tape measure, roll of painter's tape, four meters of fabric, pencils, and paper. Students were also supplied with samples of customized thermoplastic masks used for head and neck treatments. The thermoplastic masks were used as examples of immobilization and localization equipment used clinically, though students were instructed that the masks would not likely prove useful for this activity. Photographs of the activity's supplies are included in Fig. 1.

Students were asked to choose one student to act as a patient who would receive radiation therapy treatment for bony metastases to the right and left tibia. Together the groups brainstormed how to complete the entire activity. First, the “patients” were instructed to position themselves on a classroom table surface that would represent both the CT scanner table surface as well as the linear accelerator couch surface. Students were encouraged to use any tools available to them to delineate their group's patient's position. They were reminded to be respectful of their patient and the host institution's facilities. After a specified amount of time, the patient was asked to leave the treatment position carefully, walk across the room, and then return to their group's table. The group then arranged their patient back in the original position, and estimated how closely (in distance) their group was able to achieve the original position in the region of interest. A worksheet guided students through the process, though student groups ultimately decided together how to complete the initial positioning, how to reproduce that positioning, and how to evaluate their work in a project‐based learning format. Following the activity, the responsibilities that physicists hold in the CT simulation process were discussed, such as offering expertise on patient setup; designing new procedures; and acceptance testing, commissioning, and routine quality assurance of CT scanners.

### 2.B Treatment planning activity

**Figure 2:**
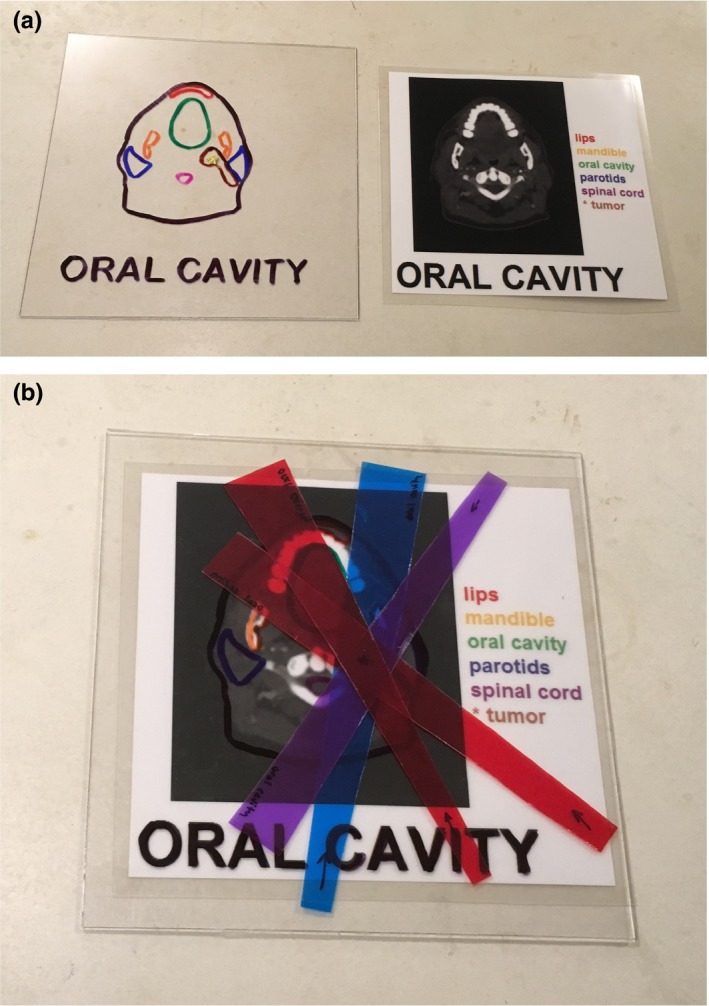
Materials used for the treatment planning activity. In (a), the oral cavity case is shown with the acrylic sheet (left) traced with color‐coded anatomical structures. The isocenter is marked with a gold star. The corresponding printed copy (right) of anonymized patient computed tomography data includes a list of target and avoidance anatomical structures, with the target structure marked with an asterisk. In (b), an example of a possible beam placement geometry is demonstrated, with transparency beams overlapping over the target region and healthy tissue avoided where possible.

The second activity examined the treatment planning process. Acrylic sheets and printed paper copies of enlarged, anonymized patient CT images (eContour, University of California‐San Diego) were used. Trapezoidal shapes were cut from colored transparency material to represent therapeutic radiation beams. The isocenter was delineated on the transparency beams as well as on the acrylic sheets. Anatomical structures were traced on the acrylic sheets using color‐coded markers to indicate both cancerous target volumes (to be “treated” using radiation) and healthy anatomical volumes (to be avoided with radiation). When the acrylic sheet is held to the light, areas where the transparencies overlap allow the least light to penetrate and appear the darkest, representing regions of high radiation dose. Students did not need to know the position or function of the different anatomical structures in order to practice basic beam placement geometry for different treatment sites. Photographs of the materials used for the activity are included in Fig. 2. Following the activity, examples of the roles physicists play in this process were discussed, including treatment planning (especially for special procedures), supporting the clinical dosimetry team, reviewing patient treatment plans, and quality assurance of the planning system.

## 3 RESULTS

A 45‐min workshop was repeated throughout University of Washington‐Bothell's 2018 Inspire STEM Festival as one of 18 workshops available to student groups. A single 3‐h workshop was conducted for the Burke Museum's middle school Girls in Science Program during the 2018–2019 school year cycle as one of a series of eight keynote educational activities. Between the two programs, approximately 130 students participated in the activities. The majority of students identified as female and attended public schools in Washington state in King and Snohomish counties. In 2018, over 600 students attended the Inspire STEM Festival, and 18 students enrolled in the Girls in Science Program. Students’ experience with the two activities is outlined below.

### 3.A Immobilization and localization activity

**Figure 3:**
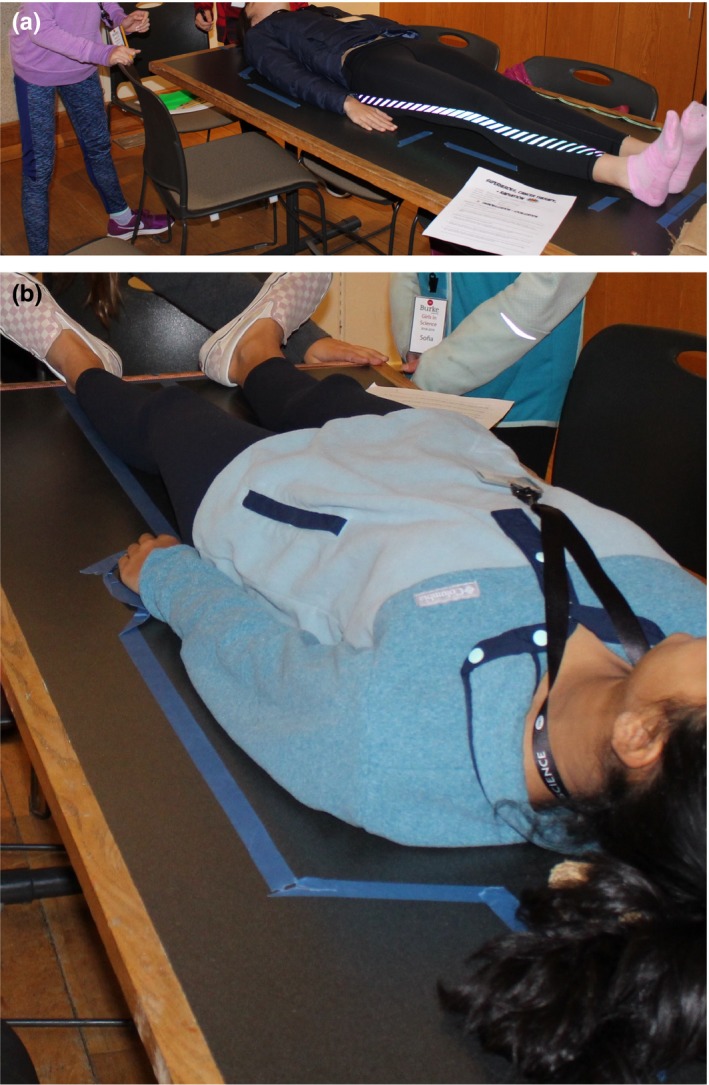
Student groups collaborating on patient setup during the immobilization and localization activity, for (a) the initial positioning phase of the activity and (b) the final verification phase of the activity.

Students were asked to decide, as a group, what would be the best way to ask a patient to position herself in order to take a CT scan of her shins. All students chose to ask their patient to lie down in the supine position on the table surface, and some groups requested that their patient remove her shoes. All groups created an outline of the patient on the classroom table surface using painter's tape. After the imaging (initial) positioning session concluded, and the patient returned for the treatment (second) alignment, students were asked to decide what region of their patient was the best aligned to its original position, and to quantify the accuracy of this alignment (in cm). For the imagined bilateral tibia treatment, most students focused on the lower legs and estimated an accuracy between 1 mm and 2 cm. Photographs of student teams working on the project are included in Fig. 3. The student worksheet included a prompt requesting that groups suggest ideas or designs for optimal immobilization equipment to use for reproducibly positioning a patient undergoing radiation therapy to the lower legs. Several teams responded by describing a foam or clay mold. During the in‐class discussion, student responses were validated by describing similar devices that are widely commercially available for use in radiation therapy, including vacuum cushions that are fitted to patients, such as the Vac‐Lock^™^ (CIVCO Radiotherapy, Orange City, IA) and polyurethane foam casts that are custom‐molded to patients, such as Alpha Cradle^®^ (Smithers Medical Products, Inc., North Canton, OH).

### 3.B Treatment planning activity

**Figure 4:**
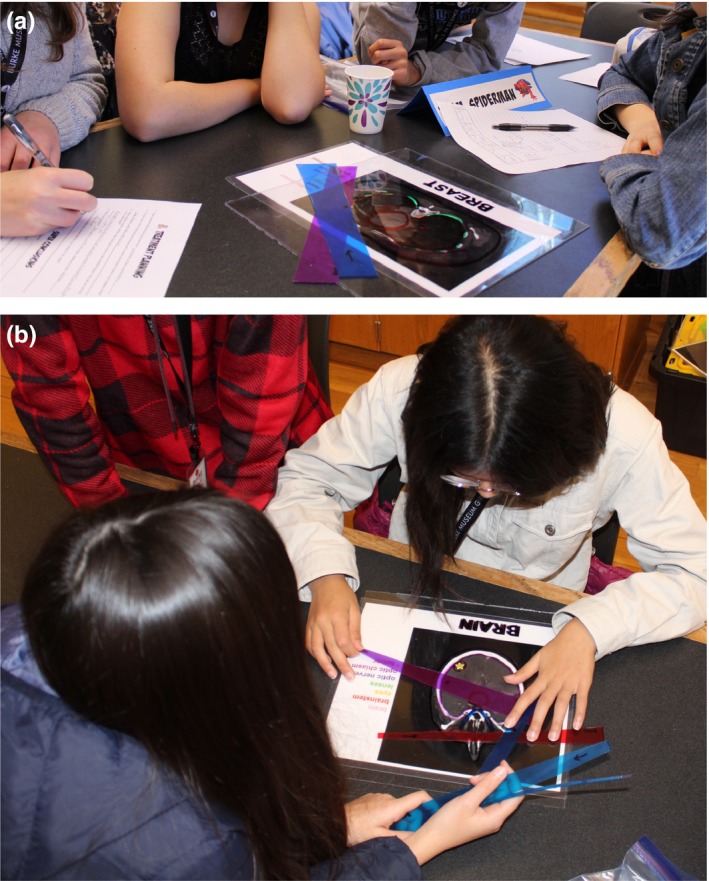
Student groups collaborating on beam placement geometry in the treatment planning activity for (a) a breast case, and (b) a brain case.

Student groups were supplied with materials to design a treatment irradiation geometry for a single treatment site. Each small group worked with a different cancer site treated using radiation, including brain, breast, esophagus, testicle, larynx, oral cavity, pancreas, and prostate. Midway through the activity, each student group traded materials with another group and discussed beam placement strategies among themselves. Photographs of students engaging with the materials are included in Fig. 4. All student groups successfully identified strategies to include the target region in their treated area, while spreading out dose to healthy tissue and avoiding critical structures when possible. In their worksheet responses, students commented on anatomical structures that should not be irradiated, and in the wrap‐up discussion, students speculated about possible risks involved in irradiating anatomical structures listed on their group's materials. Students identified required compromises as well as challenges involved in irradiating given target geometries.

### 3.C Student reflections

To conclude the activities, students were asked verbally as well as in a written survey to compare their knowledge of medical physics and radiation therapy before participation in the activities to after participation. Although most students had not heard of medical physics prior to participation, 100% of students responded via the survey that they were aware of the field following the activities. Furthermore, 75% of students responded affirmatively to the question, “after these activities, do you feel more interested in careers in physics, engineering, or medicine?” Students who participated received certificates of achievement indicating they qualified as medical physicists in training.

Secondary feedback from parents (who were not in attendance) was positive, with observations including, “This new topic […] was reported ‘amazing’…it's a yes to add for next year,” “she learned about a job she'd never even thought of,” and “the activities were A++.” One parent's assessment included paraphrased comments from the student participant: “It was so cool. […] What we learned was so amazing. It was all about radiation for cancer treatment. How to plan, measure, and deliver the radiation. It was like being at the world's fanciest private school.”

## 4 DISCUSSION

Though diverse organizations, including in science and engineering, tend to outperform their more homogeneous counterparts,^15^, ^16^, ^17^ only about half of U.S. scientists and engineers working in STEM fields identify as a classification other than white men.^1^ Over the time period from 2013 to 2017, the American Physical Society reported that fewer than 3% of physics bachelor's degrees were awarded to African Americans, and fewer than 9% to Hispanic Americans. Fewer than 20% of physics Ph.D. degrees were awarded to women, with the percentage of women earning bachelor's degrees in physics declining.^18^ A study by Georgetown University Center on Education and the Workforce^19^ noted that current underrepresentation of minority and women candidates in STEM fields represents a failure to recruit from over half the available talent pool. Reaching children at a young age in both informal and formal learning environments, especially children who may not have access to many role models in STEM, encourages sustained student engagement in STEM — and may help address this issue.^20^, ^21^ A joint report by the National Academy of Sciences, National Academy of Engineering, and the Institute of Medicine urgently advocated for raising awareness of STEM careers through activities for K–12 students, and for promoting STEM outreach efforts that are specifically aimed at members of underrepresented groups in STEM fields.^22^

Approximately 1.7 million new cancer cases occurred in the United States in 2018,^23^ and approximately half of all cancer patients receive radiation therapy during their course of treatment.^24^ Though millions of patients benefit from their work, medical physicists often remain behind the scenes, and even many patients receiving radiation therapy are not familiar with the physicists contributing to their care.^25^, ^26^, ^27^ It is therefore unsurprising that many students are not knowledgeable about the career opportunities related to this field: when surveyed high school students were asked to list three possible careers at a hospital, many students struggled to identify options beyond nurses and doctors.^28^ Targeted outreach efforts may help address this unawareness. Many medical physicists find their field to be rewarding, with the majority of surveyed physicists expressing satisfaction with their jobs and reporting a feeling of accomplishment derived from their careers.^29^ As a challenging and rewarding career choice, medical physics may be used as an example of possible future opportunities to students who previously may have never heard of the field, including members of student groups traditionally underrepresented in STEM. The activities described here are inexpensive and straightforward to implement and may be adapted and used for further outreach applications by interested physicists.

### 4.A Future work

Though UW‐Bothell's Inspire STEM Festival has been discontinued, future work will include delivery of these lesson plans again for the Burke Museum's Girls in Science Program in the 2019–2020 school year. In addition, these workshops will be delivered for a third program: the Girls in Engineering, Math, and Science (GEMS) Program through the Seattle Chapter of the Association for Women in Science. GEMS offers free, STEM‐based after‐school programming for female‐identified students in grades 7–8 enrolled in Seattle Public Schools. GEMS after‐school sessions are designed to encourage students to work in groups to complete hands‐on, workshop‐based activities. Because the intended audience and classroom format are similar across the three programs, it is expected that the lesson plans described here will require minimal revision prior to delivery at GEMS.

## 5 CONCLUSION

Detailed lesson plans were developed using two learner‐centered, active‐learning educational activities giving an introduction to careers in medical physics and radiation therapy. The lesson plans were designed to accommodate a middle school learning audience, using low‐cost or donated equipment. The workshops were successfully delivered at two different educational outreach programs, for students in grades 6–8. A third program will be added in future work. Feedback from these learning units indicate students had positive experiences and increased interest in careers in medical physics. These lesson plans are available upon request for educators interested in exploring medical physics educational outreach activities in their communities.

## CONFLICT OF INTEREST

There are no relevant conflicts of interest to disclose.
